# Crossing the comfort zone: rabbit appeasing pheromone enhances acclimation to a novel environment

**DOI:** 10.3389/fvets.2026.1929577

**Published:** 2026-08-13

**Authors:** Umut Burak Agan, Mattis Macq, Estelle Descout, Marcel Bassayi Abalo, Alessandro Cozzi, Manon Chasles

**Affiliations:** 1 Ethology, Physiology and Chemical Communication Department, IRSEA, Research Institute in Semiochemistry and Applied Ethology, Quartier Salignan, Apt, France; 2 Ethics, Legislation and Animal Welfare Department, IRSEA, Research Institute in Semiochemistry and Applied Ethology, Quartier Salignan, Apt, France; 3 Statistics and Data Management Service, IRSEA, Research Institute in Semiochemistry and Applied Ethology, Quartier Salignan, Apt, France

**Keywords:** acclimation, appeasing pheromone, coping, Open-Field (OF), rabbit

## Abstract

Appeasing pheromones are known to help animals cope with challenging and stressful situations, facilitating acclimation to diverse environments and contexts across a wide range of species. As prey animals highly sensitive to environmental stressors, rabbits are particularly good candidates to benefit from such interventions and represent a relevant species for research in this field. This study evaluated the behavioral effects of topical skin application of Rabbit Appeasing Pheromone (RAP) in rabbits exposed to a novel environment, using an Open-Field (OF) test conducted over two consecutive days. Twenty-eight rabbits were randomly divided into two groups, balanced for sex, neutering status, and parity. Behavioral parameters were assessed using both behavioral observation software and automated tracking software to determine the effects of RAP on locomotion, exploration, and grooming. During the first Open-Field test (OF1), all rabbits exhibited a behavioral profile characterized by increased locomotion. During the second exposure (OF2), this pattern shifted toward reduced locomotion and increased stationary behaviors. Rabbits exhibited increased grooming frequency during OF2, regardless of treatment. Grooming duration showed a numerical increase in the placebo group during OF2; however, this increase did not reach statistical significance. The treatment effects were most evident in measures of explorative behaviors, with RAP-treated rabbits showing faster entry into the central zone and spending more time there in both tests. In addition, a statistical tendency was observed for RAP-treated rabbits to enter the arena voluntarily sooner than placebo-treated rabbits. Overall, these results highlight the substantial role of novelty-driven locomotor activity in rabbits, along with the distinct behavioral effects of RAP on their interaction with the novel environment. In particular, the spatial use of the arena by RAP-treated rabbits across both tests is consistent with reduced anxiety-related responses to a novel environment and its repeated exposure. These findings support the potential role of topical RAP application in facilitating rabbits’ behavioral acclimation to novel and potentially challenging situations.

## 1 Introduction

Appeasing pheromones, naturally released by mothers during the neonatal period, play a critical role in reducing stress and promoting a sense of security in offspring (1). Synthetic analogs of these pheromones have been shown to improve stress coping and reduce anxiety-related behaviors not only in young but also in adults across a wide range of animal species (1–3). Given their broad efficacy, considerable efforts are underway to develop and evaluate these compounds for application across diverse animal species, contexts, and environmental settings.

Rabbits (*Oryctolagus cuniculus*), a prey species highly sensitive to environmental conditions, are widely kept in modern society as companion animals, farm animals, and laboratory models (4–6). Their growing popularity, particularly as companion animals, has made them one of the most commonly kept domestic species worldwide (7). This widespread presence across diverse settings underscores the importance of ensuring optimal welfare standards for this species. However, rabbits’ marked sensitivity to environmental challenges makes them susceptible to stress-related problems. Environmental stressors can significantly alter both behavioral and physiological parameters, thereby directly compromising welfare in this species (8, 9). In particular, exposure to novel environments, social isolation, and human handling are known to elicit marked stress responses in rabbits (9–11). Therefore, the development of new tools and practices aimed at improving stress coping and acclimation to these challenging situations has become an important area of research. In this context, the use of appeasing pheromones in this species, together with the development of practical administration methods, represents a promising approach for improving acclimation and welfare. Indeed, studies in rabbits have shown that Rabbit Appeasing Pheromone (RAP), administered through various delivery systems such as passive diffusers and mists, may effectively support coping with a range of stressful situations, including routine veterinary visits, vaccination-related procedures, and transport (12, 13). Furthermore, the application of RAP under farm conditions has been associated with easier handling of does and a trend towards improvements in reproductive parameters, such as fertility, yielding promising results (14). Although RAP has shown beneficial effects during specific acute stressors, its effects on acclimation and behavioral responses to repeated exposure to a novel environment under standardized and controlled conditions remain largely unexplored. As a widely used test, the Open-Field (OF) assesses fearfulness, anxiety-like behaviors, and associated locomotor activity (15–17). In the OF test, increased use of the central area is generally associated with lower levels of anxiety, whereas a tendency to remain close to the walls (thigmotaxis) is considered an indicator of heightened anxiety levels (17). However, behaviors observed in the OF are multifactorial and may reflect several underlying motivations, including factors such as fear-related responses, safety optimization, and exploratory drive (18–20). Importantly, behavioral responses in the OF are known to change with repeated exposure (19) allowing behavioral changes and acclimation to novelty to be evaluated over time. Despite this, the effects of RAP in a repeated OF paradigm remain largely unexplored, and evidence regarding its topical skin application is limited to one pilot study (21).

Therefore, the present study aimed to assess the effects of topical RAP (SecureBunny® Adventure, SIGNS Labs) on the behavioral responses of adult rabbits during repeated exposure to a novel environment using a standardized OF paradigm. Specifically, we investigated whether RAP could facilitate behavioral acclimation and improve coping responses across consecutive OF exposures.

## 2 Materials and methods

This experimentation was performed in accordance with the European directive 2010/63/EU on the protection of animals used for scientific purposes. The study protocol was approved by the French Ministry of Research and an external ethics committee, CEEA-07 (APAFIS number: 51821-2024101709366647 v3).

### 2.1 Animals

A total of 28 adult New Zealand rabbits aged 12 to 16 months old, were involved in this experiment. Only healthy rabbits that were able to express all behavioral patterns, as well as present normal locomotion, were included. This population included 2 intact males, 10 castrated males, 10 intact females, and 6 sterilized females. Among the 16 females, 5 were primiparous, and 11 were nulliparous. None of the rabbits had previously been subjected to an Open-Field test or exposed to the arena used in the present study, ensuring that the testing environment was novel to all subjects. Those 28 rabbits were divided into two groups balanced for their sex, neutering status, and parity. Each group was housed in a separate but similar room to prevent any potential cross-contamination during exposure to the pheromonal treatment. Except for the two intact males, all rabbits were housed in social groups of 2–3 individuals in 3 m^2^ pens on the floor. In accordance with UFAW (Universities Federation for Animal Welfare) and RSPCA (Royal Society for the Prevention of Cruelty to Animals) recommendations, intact males were housed individually but with visual, olfactory, and auditory contact with other rabbits (22). All pens contained sufficient shelters to allow all animals to use them simultaneously and were provided with ad libitum water and hay. In addition, each rabbit received a daily dose of 150 g of concentrate (Kliba-Nafag, SERLAB). Animals were closely monitored by animal keepers and veterinarians to ensure their health and ability to participate in the study. Additionally, they were monitored monthly by the Animal Welfare Body of the Research Institute (23).

### 2.2 Experimental design

Our study aimed to characterize the impact of exposure to RAP under the commercialized form SecureBunny® Adventure on the behavior of rabbits while subjected to an unknown environment. We decided to perform OF tests as it is a well-characterized test that enables us to assess the exploratory behavior of rabbits, but also as it is known to assess fearfulness and anxiety (15–18). An *a priori* power analysis based on data from previous studies conducted at our institute indicated that, depending on the outcome considered, 22–36 rabbits would be required to achieve 80% statistical power. Based on these estimates and the number of animals available for inclusion, 28 rabbits were included in the study.

For each week, we followed the same schedule: application of the treatment on the first day, followed by Open-Field tests on the two subsequent days. Subjects were randomized using the R Statistical language [version 4.4.2; R Core Team (36)]; two potential confounding factors, sex and sterilization status, were considered during randomization, and four strata were defined to ensure balanced allocation between treatment groups. For females, parity (nulliparous or primiparous) was additionally considered. A general treatment randomization list was generated to ensure random allocation of rabbits to treatment groups. Four additional randomization lists were generated, two for each study week. The study was conducted over 2 weeks, with half of the rabbits tested during the first week and the remaining half during the second week. Within each week, each rabbit underwent two tests (OF1 and OF2), with the order of testing varied across testing days to minimize potential order effects. The experimentation was blinded, and animals received either the RAP treatment (*n* = 13) or a placebo (*n* = 15); both treatments were applied by the same person. RAP and placebo were coded as Treatment A and Treatment B. The allocation corresponding to each code was known only to the chemist responsible for preparing the solutions. Blinding was maintained until the completion of all statistical analyses; operators, investigators, and data analysts remained blind throughout the study.

The RAP treatment (SecureBunny® Adventure, SIGNS Labs), a commercially available product, and the corresponding placebo were provided by SIGNS Labs. According to the manufacturer’s instructions, 1 mL of the RAP treatment or placebo was applied topically to the skin. The fur was gently parted along the back of the neck, and the tip of the tube was positioned between the bases of the ears. The cream was then applied directly to the exposed skin in a continuous line along the back of the neck, without rubbing. The RAP treatment contained 3% of a synthetic analog of the RAP in an aqueous emulsion, while the placebo was constituted of the same excipients without the active compound. The treatment was administered once, as its effects are reported by the manufacturer to persist for up to 5 days following a single application. On the first and second days following treatment administration, the animals were subjected to the experimental setting. Testing was performed at the same time of day throughout the experimental period. On each test day, each rabbit was individually transported in a cage to the experimental room where a 3 × 3 m OF arena had been set up. The 0.80-m-high walls of the OF arena were covered with opaque fabric to prevent visual contact with the surroundings, and the floor was lined with a non-slip mat to ensure proper locomotion. The transport cage was placed on the floor and gently opened in front of the closed sliding door of the arena. After a 1-min acclimatization period, the sliding door was opened, allowing the rabbit to enter the arena voluntarily within 1 min. If the rabbit stayed inside the transport cage after this period, the operator gently encouraged it to enter the arena using the cage’s push panel. Once all four legs of the rabbit were inside the arena, the sliding door was gently closed, and a 5-min timer was started. At the end of the test, the animal was returned to its home pen, and the arena was cleaned.

### 2.3 Behavioral analysis

Rabbits’ behavior was analyzed for 5 min, starting when the rabbits had fully entered the arena and the door was closed. During all tests, a GoPro Hero 9 was mounted on the ceiling directly above the center of the arena. Videos were anonymized and analyzed by two independent readers trained in animal behavior observation using BORIS (Behavioral Observation Research Interactive Software) (24).

The rabbit ethogram has been established according to previously published studies investigating rabbit behavior in an Open-Field device (15). In our study, all behaviors were divided into three categories (Table 1), and they were either analyzed as frequency (f), duration (d), or latency (l). For latencies, in the case a rabbit never reached the criteria, the maximum time duration was applied (Table 1).

**Table 1:** Description of the behaviors observed during the video analysis.

Category	Behavior	Description	Software used
Exploration	Voluntary entry (l)—maximum latency 60s	The rabbit enters the arena with its four legs within 1 min after the door is opened	Boris
	Central area (l, f, d)—maximum latency 300 s	The rabbit enters the central area of the Open-Field (Figure 1)	AnimalTA
	Percentage of arena explored	Percentage of the arena that has been visited at least once by the rabbit	AnimalTA
	Environment investigation (d)	The rabbit is smelling the floor or walls of the Open-Field arena	Boris
Locomotion	Distance	Total distance covered in meters	AnimalTA
	Hop (d)	The rabbit is moving, forelegs are moved alternately, while hind legs are moved synchronously	Boris
	Walk (d)	The rabbit is moving, forelegs as well as hindlegs move alternately	Boris
	Stay (d)	The rabbit is not moving; both fore and hind legs are on the ground	Boris
	Partial move (d)	The rabbit moves only its front legs	Boris
Other	Grooming (f, d)	The rabbit displays self-directed cleaning behavior	Boris

f, frequency; d, duration; l, latency.

### 2.4 Tracking analysis

**Figure 1:**
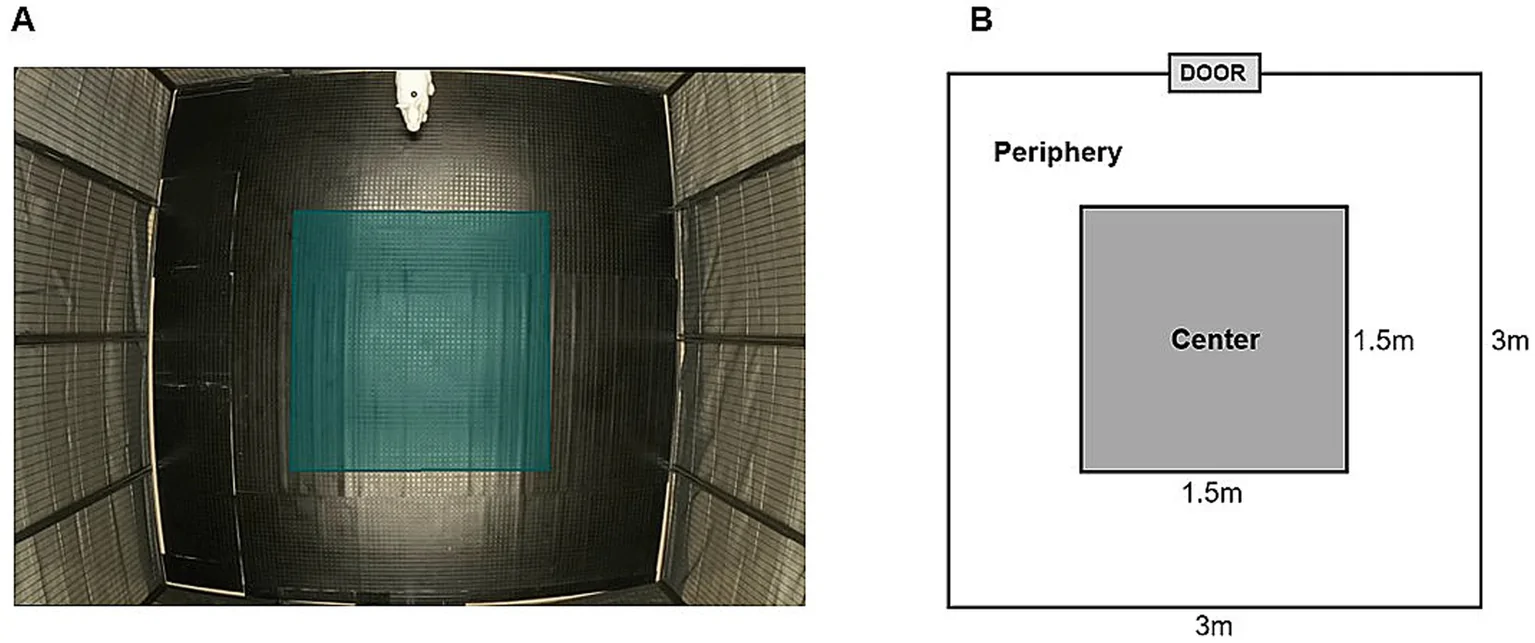
Picture **(A)** and scheme **(B)** of the experimental setting showing the central area. Animals entered the 3 × 3 m Open-Field by a sliding door at its top. The test lasted for 5 min. To analyze the time spent at the center of the Open-Field arena, a square of 1.5 × 1.5 m was drawn in the AnimalTA software.

**Figure 2:**
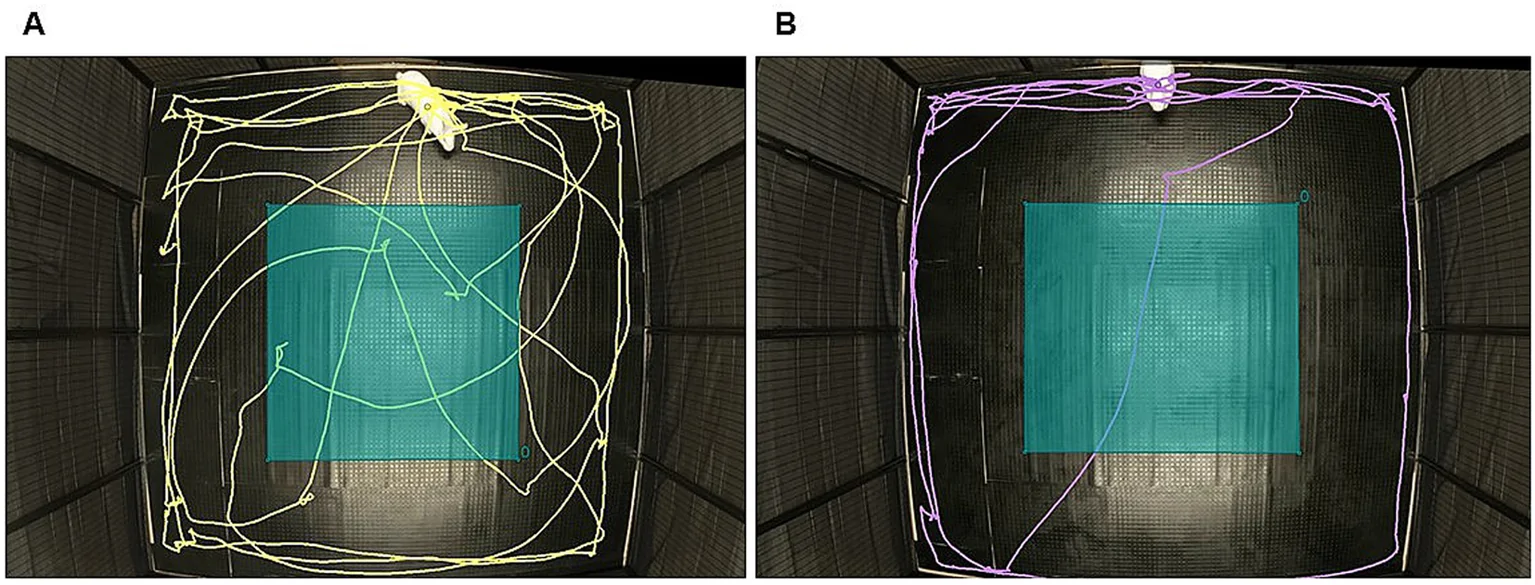
AnimalTA-generated trajectories showing arena space use by a rabbit from the RAP treatment group **(A)** and a rabbit from the placebo group **(B)**.

In addition to the classical behavioral analysis described above, we performed an automatic tracking analysis using the AnimalTA (25) software to characterize the animals’ trajectories. As a key parameter of the OF test is the time spent in the center compared to the periphery, a central area of 1.5 × 1.5 m was defined using the spatial repartition analysis menu, allowing the addition of “elements of interest” (Figure 1). Using the exploration menu of the software, we defined an “interest surface” of 2,500 cm^2^ centered on the body of the rabbit, representing the explored area around the animal. Using this feature, we were able to estimate the percentage of the arena explored by the animal. Moreover, as the dimensions of the Open-Field arena were known, we could specify the scale, 3.25 pixels per cm, enabling us to quantify the total distance covered by each animal in centimeters (Table 1). The tracking software also enabled visualization of the individual trajectories of each rabbit (Figure 2). As the camera was mounted on the ceiling directly above the center of the arena, animals were always detectable, and no shadows could interfere with the tracking; thus, there was no lost data in the tracking analysis.

### 2.5 Statistical analysis

The data were analyzed using R [version 4.5.0; R Core Team (37)] with RStudio 2025.05.0 + 496. The significance threshold was fixed at 5%. For video analysis, inter-observer reliability between the two independent readers was assessed for each parameter. Normality was evaluated using the Shapiro–Wilk test and QQ plots. Pearson or Spearman correlation coefficients were calculated depending on normality. The reader association was obtained by squaring the correlation coefficient. If the association exceeded 80%, the mean (continuous variables) or maximum (discrete variables) value was used for further analyses; otherwise, the variable was re-evaluated until agreement was reached. All models included treatment (RAP or Placebo), test (OF1 or OF2), and their interaction as fixed effects, and rabbit ID, cage, week, and neuter status as random effects. The final model was obtained by simplifying the models when the AIC and BIC criteria decreased after all non-significant terms were removed. The significance of parameters was then assessed using the Likelihood Ratio (Chi-square χ^2^) test (CAR package). *Post-hoc* pairwise comparisons (Tukey-adjusted) of estimated marginal means were performed on factors with more than two levels (EMMEANS package).

Behavioral latencies were analyzed using time-to-event survival analysis (Coxme package). The condition of proportional hazard was verified and validated with the study of Schoenfeld residuals. Behavior frequencies were analyzed using generalized linear mixed models (GzLMM) with Poisson distribution and log link function (lme4 package). The dispersion of the resulting model was assessed using Pearson chi-square/DF statistics. The variable representing the percentage of arena explored was analyzed using a GzLMM with a Beta distribution and a logit link function (glmmTMB package). Variables related to durations of behaviors and distance covered in the arena were analyzed with general linear mixed models (GLMM) using the lme4 package. The model residual conditions of normality and homoscedasticity were verified and validated, except for grooming duration, where excessive zeros were detected in the data; a zero-inflated hurdle log-normal was performed (GLMMadaptive package).

## 3 Results

### 3.1 Grooming behavior

**Figure 3:**
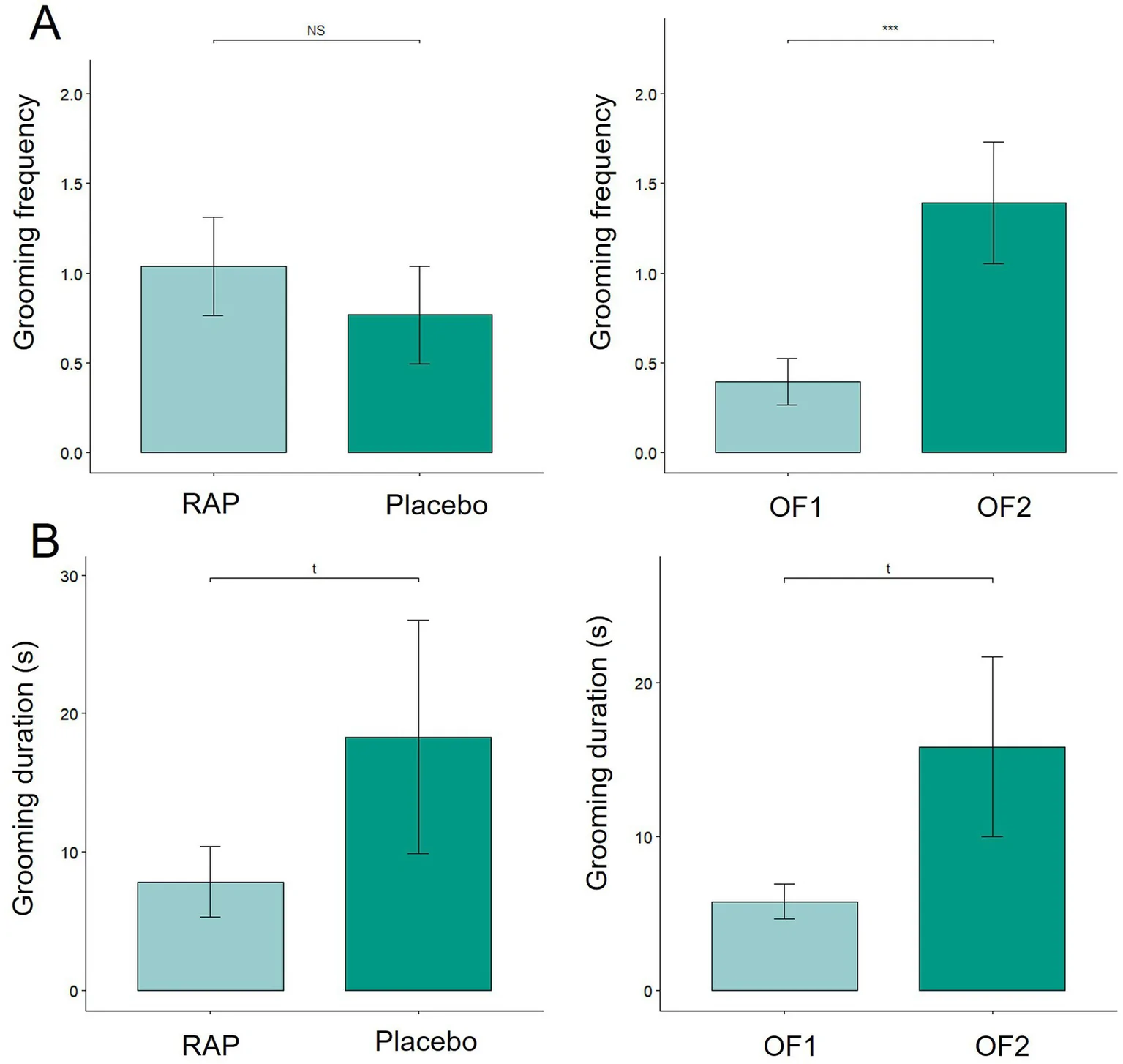
Grooming frequency **(A)** and duration **(B)** across treatment groups (RAP and placebo) and test sessions (OF1 and OF2). Grooming frequency was analyzed using a Generalized Linear Mixed Model with a Poisson distribution, while grooming duration was analyzed using a zero-inflated General Linear Mixed Model with a hurdle log-normal distribution. s, seconds; NS, non-significant; t, tendency; ***p < 0.001. Error bars correspond to the standard error.

Regarding grooming frequency (Figure 3A), we did not detect any treatment effect (GzLMM, DF = 1, *p*-Value > 0.05), but a strong test effect was observed (GzLMM, DF = 1, *p*-value < 0.001) with rabbits grooming more frequently during OF2 compared to OF1. For grooming duration (Figure 3B), neither the treatment effect nor the test effect reached statistical significance, although a statistical tendency was observed (lognormal hurdle model, positive component, *p* = 0.07 and *p* = 0.06, respectively), with grooming duration higher in the Placebo group and during OF2.

### 3.2 Explorative behaviors

**Figure 4:**
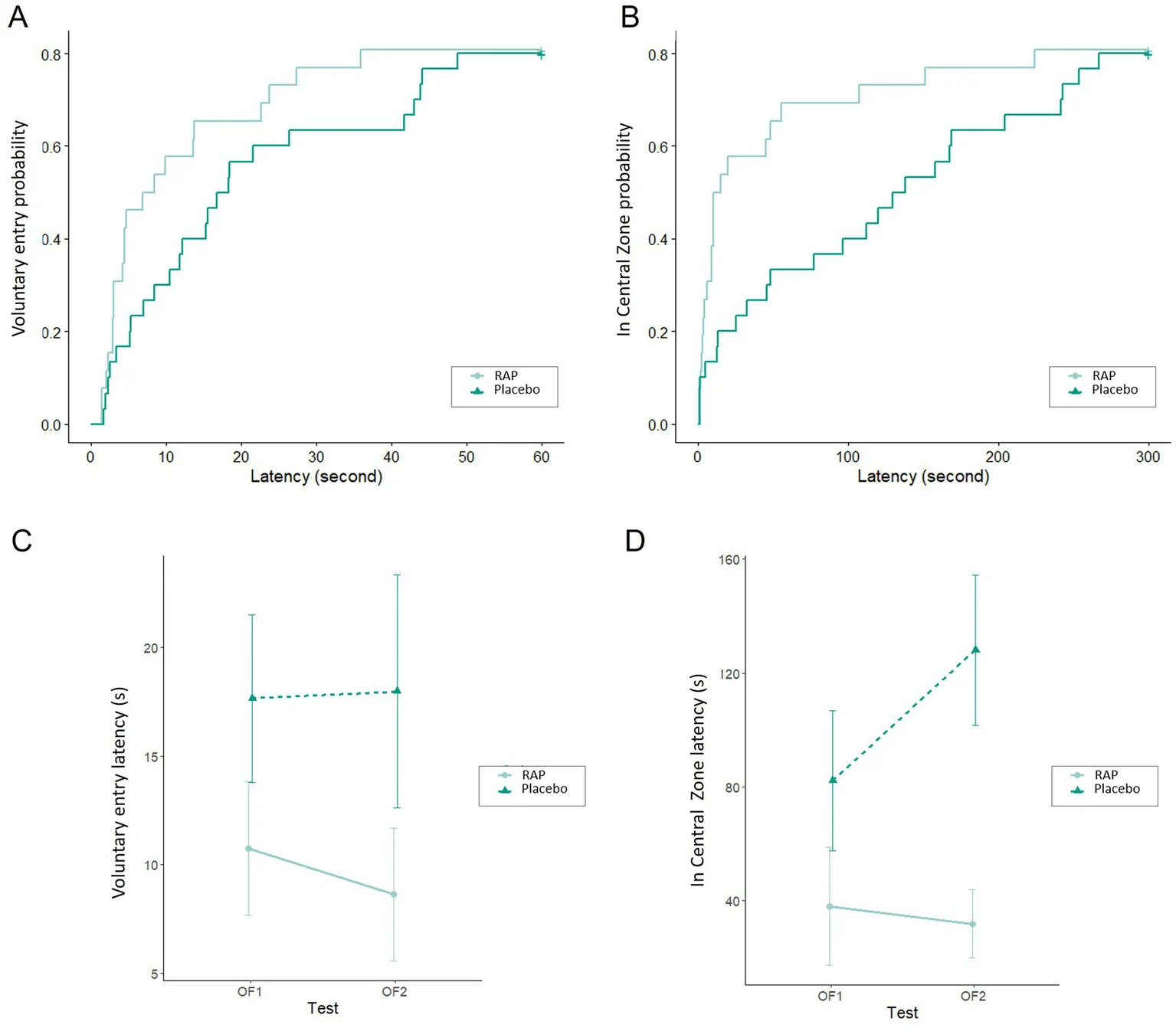
Survival curves illustrating treatment effects on event risk for voluntary entry **(A)** and center zone entry **(B)**, and line plots showing mean latency to voluntary entry **(C)** and center zone entry **(D)** across test sessions (OF1, OF2) for each treatment group. The effect of treatment on the relative risk of voluntary entry latency and center zone entry latency was analyzed using a Cox frailty model. Error bars correspond to the standard error. s, seconds.

For the latency to voluntarily enter the arena (Figures 4A,C), the treatment effect alone was not significant (Cox-frailty, DF = 1, *p* > 0.05), but we observed a significant effect of the test, with rabbits entering the arena more quickly during OF2 (Cox-frailty, DF = 1, *p* < 0.05, Figure 4C). A statistical tendency for treatment x test interaction effect was also found (Cox-frailty, DF = 1, *p* = 0.06).

**Figure 5:**
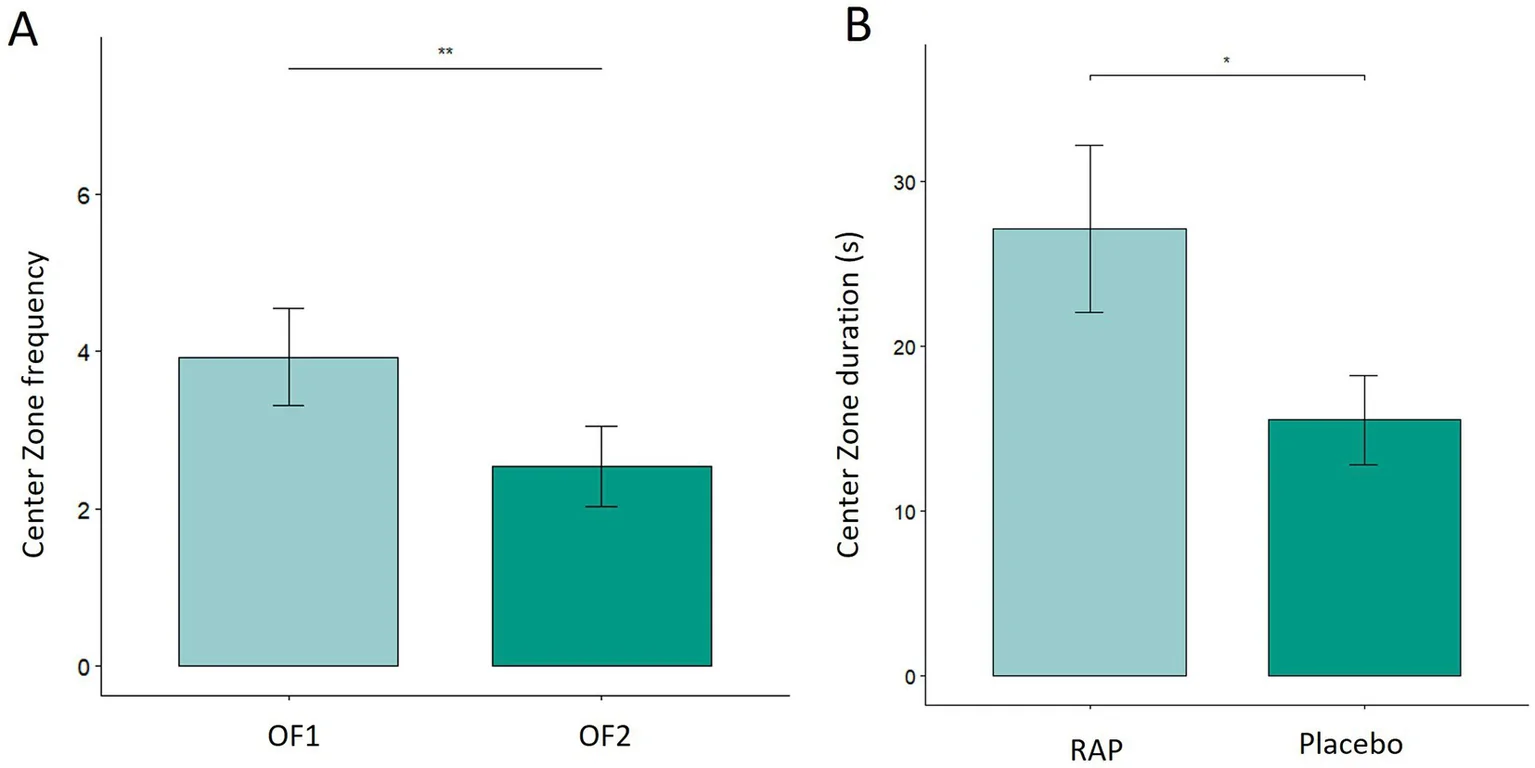
**(A)** Frequency of entries into the center zone across test sessions (OF1 and OF2), and **(B)** time spent in the center zone across treatment groups. Center zone entry frequency was analyzed using a generalized linear mixed model with a Poisson distribution, while center zone duration was analyzed using a general linear mixed model. s, seconds, *p < 0.05; **p < 0.01. Error bars correspond to the standard error.

Regarding the latency to reach the central zone (Figures 4B,D), we found a test effect, as rabbits were significantly quicker to reach the center during OF1 (Cox-frailty, DF = 1, *p* < 0.05, Figure 4D). Besides, a treatment effect was found, as RAP-treated rabbits reached the center more quickly than the Placebo-treated ones for both tests (Cox-frailty, DF = 1, *p* < 0.05, Figure 4D). A statistical tendency for treatment x test interaction effect was also found (Cox-frailty; DF = 1, *p* = 0.07). No treatment (GzLMM, DF = 1, *p* > 0.05) effect but a test (GzLMM, DF = 1; *p* < 0.01) effect was detected for the frequency of entry into the central zone (Figure 5A). However, a treatment effect was observed for the time spent inside the central zone (Figure 5B), with RAP-treated rabbits spending significantly more time in this area (GLMM, DF = 1, *p* < 0.05).

The percentage of the arena explored by each rabbit has been characterized using the tracking software; no effect of the treatment could be observed (GzLMM, DF = 1, *p* > 0.05), while rabbits explored the arena less in OF2 than in OF1 (GzLMM, DF = 1, *p* < 0.05).

**Figure 6:**
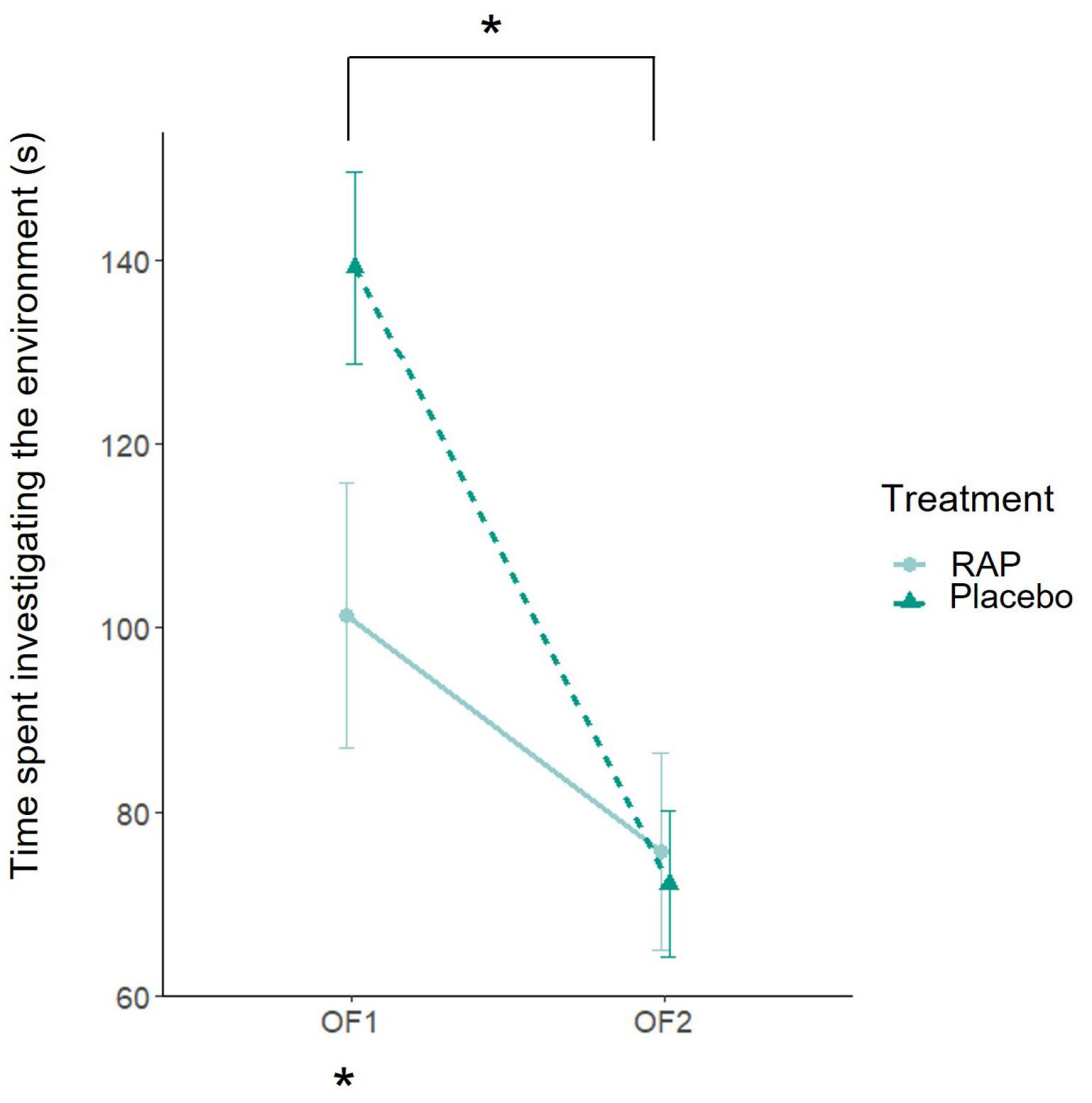
Line plot illustrating time spent investigating the environment across test sessions (OF1 and OF2) and treatment groups. Time spent investigating the environment was analyzed using a General Linear Mixed Model, revealing significant effects of treatment, test, and their interaction. s, seconds, *p < 0.05. Error bars correspond to the standard error.

For the time spent investigating their environment (Figure 6), we found a treatment effect (GLMM, DF = 1, *p* < 0.05), a test effect (GLMM, DF = 1, *p* < 0.05), as well as a treatment x test interaction effect (GLMM, DF = 1, *p* < 0.05). Multiple comparisons revealed that during OF1, Placebo-treated rabbits spent more time investigating their environment than RAP-treated rabbits (GLMM, DF = 1, *p* < 0.05) and that, in both treatment groups, this behavior was significantly decreased during OF2 compared to OF1 (GLMM, DF = 1, *p* < 0.05, Figure 6).

### 3.3 Locomotor activity

Locomotor activity has been characterized on the basis of different parameters, including the total distance (in meters) covered by animals during the test. This distance parameter did not change according to the treatment (GLMM, DF = 1, *p* > 0.05), but the distance covered was higher during the first Open-Field than the second one (OF1: 36.56 ± 12.91 vs. OF2: 28.55 ± 11.81, GLMM, DF = 1, *p* < 0.001).

**Figure 7:**
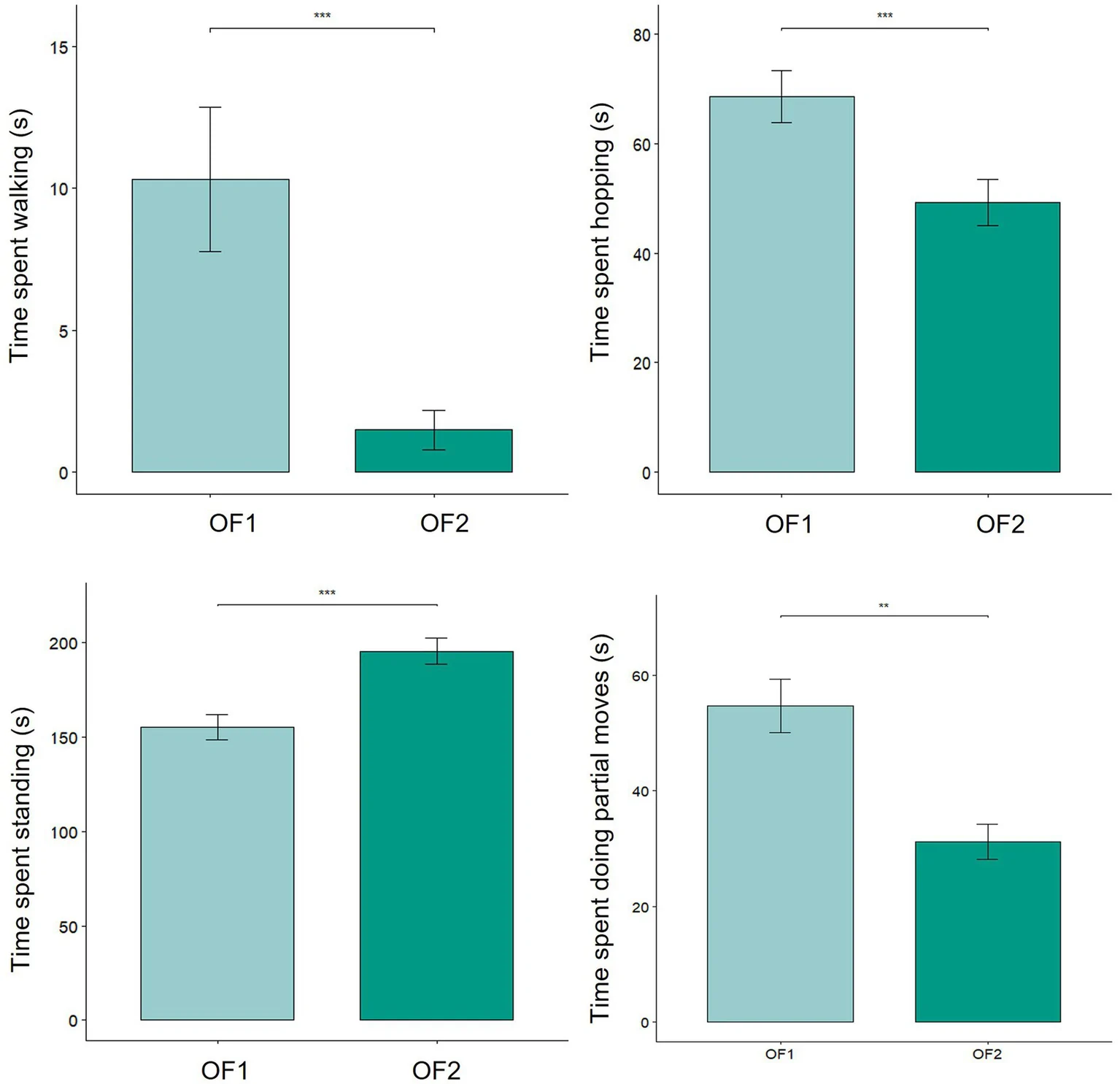
Changes in the duration of locomotor behaviors coded using BORIS across sessions (OF1–OF2). Time spent walking, hopping, and standing was analyzed using a general linear mixed model, whereas partial movement frequency was analyzed using a generalized linear mixed model with a Poisson distribution. s, seconds; **p < 0.01; ***p < 0.001. Error bars correspond to the standard error.

Regarding the time spent walking, standing, hopping, or doing partial move, no effect of the treatment was found (GLMM, DF = 1, *p* > 0.05), but a test effect was observed (Figure 7). During OF1, rabbits spent more time walking (GLMM, DF = 1, *p* < 0.001), hopping (GLMM, DF = 1, *p* < 0.001), and doing partial moves (GLMM, DF = 1, *p* < 0.01). By contrast, standing behavior was increased for OF2 (GLMM, DF = 1, *p* < 0.001).

## 4 Discussion

In the present study, we evaluated the behavioral effects of topical skin application of RAP on rabbits exposed to a novel environment using an Open-Field test setting over two consecutive days. During the first test (OF1), all rabbits exhibited a behavioral pattern characterized by increased locomotion. During the second exposure (OF2), this pattern shifted toward reduced locomotion, alongside increased stationary behaviors. Additionally, the percentage of the arena explored was lower for all rabbits during OF2. This shift suggests a strong novelty-driven locomotor activity in rabbits and highlights the importance of repeated exposure design. Treatment effects were most evident in measures of exploratory behavior, with RAP-treated rabbits entering the central zone more rapidly and spending more time there across both tests. These consistent effects suggest that RAP-treated rabbits interacted differently with their environment both under novel conditions and following repeated exposure.

Beyond these overall changes, rabbits exhibited an increased frequency of grooming in OF2, with a tendency toward longer grooming duration in placebo-treated rabbits. This pattern may reflect a shift in behavioral strategy as the environment becomes more familiar. Grooming, in this context, can be interpreted as a self-directed coping behavior, potentially indicative of stress regulation, but may also represent a maintenance-related activity associated with reduced novelty (26). Evidence suggests that grooming in rabbits may increase, particularly toward the end of a stressful event, as a compensatory mechanism (27). Thus, the observed trend toward increased grooming duration in placebo-treated rabbits may be interpreted as a self-soothing response following their first encounter with the OF test environment. Compared to RAP-treated rabbits, these animals may have experienced higher levels of anxiety when exposed to the novel environment. However, the present study did not further characterize grooming patterns to determine whether they were complete and regular or instead fragmented and irregular. Notably, stress is known to alter grooming sequences, often resulting in incomplete or disorganized grooming behavior (28). Future studies incorporating detailed analyses of grooming patterns would enable a more accurate classification of the valence of grooming behavior.

Regarding exploratory and spatial behaviors, these measures provide important insights into treatment effects. The shorter latency to enter the arena during OF2, irrespective of group, is likely attributable to habituation. However, a statistical tendency was observed across both tests, with RAP-treated rabbits exhibiting lower entry latencies than controls, suggesting a potential facilitative effect of RAP on arena entry. Although this effect did not reach statistical significance, it may become more evident with a larger sample size. This interpretation is supported by findings from a previous pilot study, in which control rabbits were less likely to enter the arena compared to RAP-treated animals (21). When considered together, these results may suggest that RAP has the potential to facilitate entry into novel and potentially anxiety-provoking environments. Nevertheless, this interpretation remains tentative and requires further investigation with a larger sample size.

The OF test is widely used to evaluate anxiety-related behavior, particularly in prey species, where avoidance of the central area and a preference for the periphery (thigmotaxis) are positively associated with increased anxiety levels (17). In this context, the greater time spent in the central zone by RAP-treated rabbits suggests a reduction in anxiety-related behavior. This interpretation is further supported by pharmacological evidence indicating that anxiogenic agents reduce central zone exploration, whereas anxiolytic compounds produce antithigmotactic effects (29, 30). In addition to these observations, the shorter latency to reach the central zone and the higher frequency of central entries observed in both groups during OF1 likely reflect an elevated exploratory drive during initial exposure to the environment. Notably, RAP-treated rabbits consistently reached the central zone more rapidly than placebo-treated animals across both tests, which may indicate a facilitative effect of RAP on acclimation to a novel environment. Taken together, these findings suggest that RAP may accelerate and facilitate acclimation to a new environment.

The present study assessed environmental investigation, primarily based on sniffing behavior directed at the floor and walls of the arena. A difference between treatments was observed during the initial exposure, with placebo-treated rabbits spending more time investigating than RAP-treated animals. As sniffing is a key olfactory exploratory behavior in rabbits, this difference may indicate decreased novelty-driven investigation or increased sense of security in RAP-treated individuals (31). This pattern is consistent with the concept of safety optimization, suggesting that exploratory behavior reflects not only exploration or fear, but also adaptive responses involving the search for secure points and resources aimed at minimizing risk in unfamiliar environments (20).

Overall, RAP-treated rabbits exhibited distinct interactions with the environment, particularly in measures of space use and initial responses to novelty. Their shorter latency to enter the central zone and increased time spent there may suggest a more positive evaluation of environmental risk, potentially reflecting reduced anxiety or enhanced acclimation to novelty. In contrast, locomotor activity appeared largely unaffected by treatment. Instead, behavioral changes across sessions suggest reduced exploration, as evidenced by greater distance covered and increased locomotor activity during OF1, followed by a shift toward more stationary behaviors, such as standing, in OF2. This pattern suggests that locomotor activity decreases as the environment becomes more familiar, likely due to a reduction in exploratory drive. These results in accordance with a previous report claiming that a reduction in locomotor activity with repeated open field testing might be more strongly related to exploratory drive rather than fearfulness (19). However, locomotor activity in a novel environment may be shaped by multiple interacting factors, including exploratory drive, fear, and safety optimization processes (18, 20, 32). Accordingly, changes in activity levels should be interpreted considering the interplay of multiple factors rather than a single underlying state.

Consistent with previous studies, our findings suggest that RAP may enhance rabbits’ acclimation to challenging situations. Prior work has shown that RAP delivered via passive diffusers reduces agitation and improves coping during veterinary procedures, as indicated by ear posture and visual analog scale measures (12). Similarly, mist formulations have been associated with improved responses to transport, possibly by facilitating adaptive coping mechanisms (13). Present results are consistent with these previous reports, particularly regarding enhanced acclimation to a potentially challenging situation. However, differences in delivery method and experimental context should be considered when comparing findings across studies.

Appeasing pheromones are thought to be detected by the vomeronasal organ, with signals relayed via the vomeronasal nerve to the accessory olfactory bulb and subsequently to limbic system structures. This pathway is considered to play a key role in the modulation of emotional processing and behavioral responses (1). Accordingly, skin application of RAP may provide continuous exposure, as the pheromone is carried by the rabbit itself and therefore remains present across indoor and outdoor environments, as well as across varying environmental and social contexts. This sustained availability may have contributed to the behavioral changes observed in the present study. Previous studies have reported pheromone-based products to be well tolerated and not associated with adverse effects (33). During the present study, the rabbits were closely monitored by the veterinary and animal welfare teams, and no adverse events or local reactions related to RAP application were observed, consistent with previous findings.

Several limitations of the present study should be acknowledged. First, the relatively small sample size may have limited the statistical power to detect certain effects of RAP, thereby increasing the risk of Type II errors. Future studies, including larger sample sizes, may provide a more comprehensive assessment of RAP-induced behavioral changes. Additionally, it is known that individual differences may affect Open-Field responses in rabbits; for example, younger rabbits may exhibit reduced locomotion and increased grooming behavior compared with older individuals (34). All rabbits included in this study were New Zealand White rabbits between 12 and 16 months of age. Although these inclusion criteria provided a more homogeneous study population, they may limit the generalizability of the findings to rabbits of other breeds and age groups. Furthermore, it is known that observable behavioral responses in rabbits may vary according to individual behavioral profile regarding their activity and lateralization (preference for right- or left-hand turn) (35). Therefore, it should be noted that such individual differences may have influenced the behavioral results of the present study.

## 5 Conclusion

The present study demonstrates that topical skin application of RAP influences rabbits’ behavioral responses to a novel environment. Particularly, behavioral patterns observed in both tests related to spatial use of the arena in RAP-treated rabbits suggest a reduction of anxiety-related behaviors. The observed behavioral shift between test sessions highlights the importance of repeated exposure and suggests that behavioral responses in the OF reflect a dynamic interplay between novelty, fearfulness, and safety-related behavioral strategies. The results of this study suggest that the topical skin application of RAP supports behavioral acclimation to novel environments in rabbits; however, further studies with larger sample sizes are needed to confirm and strengthen these findings.

## Data Availability

The raw data supporting the conclusions of this article will be made available by the authors, without undue reservation.
